# Evaluating 5.5 Years of Equinella: A Veterinary-Based Voluntary Infectious Disease Surveillance System of Equines in Switzerland

**DOI:** 10.3389/fvets.2020.00327

**Published:** 2020-06-30

**Authors:** Ranya Özçelik, Claudia Graubner, Franziska Remy-Wohlfender, Salome Dürr, Céline Faverjon

**Affiliations:** ^1^Veterinary Public Health Institute, Vetsuisse Faculty, University of Bern, Bern, Switzerland; ^2^ISME-Equine Clinic Bern, Vetsuisse Faculty, University of Bern, Bern, Switzerland

**Keywords:** equine, surveillance system, infectious disease, voluntary reporting, veterinary-based

## Abstract

Equine health is important in regard to trade, economy, society, and the veterinary, as well as public health. To reduce the burden of equine infectious diseases internationally, it is important to collect, review, and distribute equine health surveillance data as accurate and timely as possible. Within this study, we aimed at providing a comprehensive descriptive analysis of data submitted to Equinella, a voluntary veterinary-based surveillance system of non-notifiable equine infectious diseases and clinical signs, in Switzerland. This was achieved by reviewing the reports submitted since its relaunch in November 2013 and until April 2019, as well as assessing the data validity, activeness of participating veterinarians, coverage of the equine population, geographical representativeness, and timeliness of the system. In total, 630 reports have been submitted. Data validity ranged between 88.2 and 100%. The coverage of Equinella was assessed to be 50.8% of the Swiss equine population. Over the 5.5 years, of all 102 registered veterinarians, 67 (65.7%) submitted at least one report. On average, these veterinarians submitted 1.7 reports per year (median = 4 reports). More recently, in 2018, approximately only one-third [29 (28.4%)] of all registered veterinarians submitted at least one report. However, 59 (57.8%) have responded to the monthly reminder emails to confirm that they have not observed any relevant clinical case to be reported at least once (median number of confirmation per veterinarian = 9 of 12 reminder emails). The incidence of reports varied between cantons (member states of the Swiss confederation). The median timeliness of report submission was found to be 7 days. Overall, Equinella has been receiving reports since its initiation and contributed continuously to the surveillance of infectious diseases in the Swiss equine population and provided an output for the international equine community. Challenges encountered in achieving a higher number of submitted reports and increasing the coverage of the equine population, as well as the overall activeness of veterinarians, require further work. With our study, we provide a comprehensive overview of a veterinary-based voluntary surveillance system for equine health, assessed challenges of such, and suggest concrete improvements with transdisciplinary approaches for similar veterinary-based surveillance systems.

## Introduction

Equine health is important in regard to trade, economy, society, and the veterinary, as well as public health. In Europe, examples of most relevant equine infectious diseases are equine viral arteritis (1, 2), equine infectious anemia (3), strangles (4), and equine influenza (5). Such diseases not only affect animal health and welfare but also have the potential to disturb equestrian events, restrict movements of equines, cause veterinary care costs, and thus lead to economic losses for the equine industry (1, 5–7). Besides the economic impact, equine diseases can have a detrimental effect on the owner's emotional well-being (8, 9). Moreover, equine infectious diseases with zoonotic potential threaten the public health sector (10–13). West Nile fever is an example of a zoonotic disease of growing concern in Europe, as the number of reported outbreaks in humans and equines has been continuously increasing since 2013 (14, 15). Equine as well as human West Nile fever–infected individuals include asymptomatic cases up to severe meningoencephalitis (16, 17). In order to reduce the burden of equine infectious diseases for the equine industry and the veterinary, as well as public health sectors, it is important to collect, review, and distribute equine health data as accurate and timely as possible.

The change in use of equines from mainly livestock and working animals toward companion animals and the diversity in their use (e.g., breeding, food production, competitive equestrian sport, traditional and cultural events, companion animals, therapy of people with disabilities) resulted in a complex equine industry made of numerous stakeholders with disparate interests (18–20). This makes the surveillance and control of diseases in equines particularly challenging. In addition, the growing popularity of equestrian events in the past two decades has increasingly led to movement of live equines and trade of equine products (i.e., embryos, semen, equine meat) within and across country borders (21, 22). This increased mobility favors the spread of equine infectious diseases (22–24), highlighting the need to develop efficient surveillance systems (24–26).

To tackle the challenge of disease surveillance in equines, different surveillance strategies have been implemented in Europe. While notifiable diseases are being monitored via active surveillance and/or mandatory notification (passive surveillance), non-notifiable diseases are often poorly or not tracked at all. Most endemic diseases belong to this second category, and in most countries, it is up to the equine industry to manage them (5). To improve early detection of notifiable and non-notifiable diseases, surveillance systems based on voluntary notifications have been developed. For example, in France, the RESPE [Réseau D'épidémio-Surveillance en Pathologie Équine (the French Epidemiological Network for Equine Diseases), https://respe.net/] collects information about syndromes observed in the field by sentinel veterinarians and subsidizes laboratory testing. In the United Kingdom, the National Equine Health Survey^1^ collects disease information transmitted by equine owners on an annual basis (27, 28). These systems were proven to be valuable in supporting early detection (29) and estimating disease prevalence (27).

In Switzerland, today's equine industry encompasses ~125,000 equines (https://tierstatistik.identitas.ch/en/genus-equids.html) and was accounted for 1.9 million Swiss Francs (about 1.7 million Euros) turnover in agriculture in 2012 (19). The equine population has been growing by 4% annually between 2002 and 2012 (30). The current surveillance of equine health in Switzerland includes the mandatory reporting of notifiable diseases (Supplementary Table 1) to the veterinary authorities and actions to control the diseases in case of its occurrence according to the Swiss animal health law (31). In addition, a veterinary-based voluntary surveillance system for clinical signs and equine diseases not notifiable by Swiss law, called “Equinella^2^,” is in place. Equinella was introduced in 1990 (32) with joint efforts of the Federal Food Safety and Veterinary Office, the Swiss Association of Equine Practitioners, and the Equine Clinic of the University of Bern. The objectives of Equinella are monitoring and early detection of equine infectious diseases in Switzerland through a timely reporting of clinical signs and non-notifiable diseases, as well as the dissemination of acquired information nationally and internationally. In 2011, it was shown that Equinella's paper format reporting system was no longer representative of the Swiss equine population (33), and Equinella was relaunched in November 2013 as an online reporting and information platform (34). This new version consists of two main pillars: (i) the reporting of clinical signs and syndromes for syndromic surveillance and (ii) the reporting of non-notifiable equine infectious diseases (35). The data collected within Equinella have not been reviewed comprehensively since its online initiation in 2013.

The aim of our study was to provide a comprehensive descriptive analysis of a veterinary-based voluntary equine surveillance system, Equinella, by reviewing the reports submitted since its relaunch in November 2013 and until April 2019, as well as assessing the data validity in regard to data quality, activeness of participating veterinarians, the coverage of the equine population, geographical representativeness, and timeliness of the system.

## Materials and Methods

### The Equinella Reporting System

The new version of Equinella was launched in November 2013 and is set up as an online veterinary-based voluntary reporting system for equine clinical signs and syndromes (thereafter called clinical signs) and non-notifiable infectious diseases. Veterinarians must register to Equinella before being able to submit reports. This registration allows a login-secured procedure before a new report can be submitted. Furthermore, at the time point of the initial registration, participating veterinarians have to specify some information about the veterinary practice or clinic (further referred to as “practice”) they are working for (email contact and mobile phone number, practice name and address, number of veterinarians treating equines, number of equine specialists, percentage of equine workload, and approximate number of equine patients). This information is collected to evaluate different aspects of the surveillance system such as the coverage of the Swiss equine population and number of equine specialists participating in the system. Veterinarians submit reports resulting from observations of their daily patient visits.

Within Equinella, a report consists of the observation of one or more clinical sign(s) and/or suspected (or later by a laboratory test confirmed) diseases in one equine. In practice, however, if more than one equine is diseased on the same premise during the same visit, this number is occasionally provided in the free text field of a report. To enable a standardized data collection, data entry is managed by predefined checklists for most parameters. These include lists for clinical signs and non-notifiable diseases, the date when these were first observed (date of finding), age categories of the equine (≤ 6 months, 7 months to 4 years, >4 years, unknown age), and the number of equines on the premise (<5, 5–10, 11–20, 21–50, >50, unknown). Clinical signs and diseases that are not listed can be reported as free text. Identification of the equine [name, UELN (Universal Equine Life Number) or microchip number] is reported as free text. Information on the related premise (name of the premise, zip code, and town name) is also recorded. Date of report submission is automatically registered by the system. The reporting veterinarian has the option to supplement reports with laboratory test results (positive or negative results of suspected and previously reported disease) within 30 days after the original entry of a report. After the revision by a member of the Equinella expert team (one equine practitioner specialized in epidemiology and one equine practitioner specialized in internal medicine), reports are anonymized and published on the Equinella website in form of a continuous table (www.equinella.ch).

Monthly reminder emails are automatically sent to all registered veterinarians. In case the veterinarian has submitted a report in the past month, the reminder email includes only a list of these reports. In case a veterinarian has not submitted a report in the past month, these emails include two optional links: one to the Equinella reporting tool and one to confirm that in the past month no relevant case was observed. When selecting the latter, the system registers this information as “confirmation of no report.” When selecting the first, visiting the Equinella tool to submit a report from the past month, this action is not tracked by the system. Every month, the system tracks the activity of each veterinarian by providing three status options: “reported last month,” “confirmation of no report,” or “no confirmation” (when there was neither a report submitted in the past month nor a confirmation of this was given).

In order to promote the reporting via Equinella, non-monetary incentives are offered to registered veterinarians. These include a monthly electronic newsletter on national and international equine health events, one free professional veterinary education course per year, direct contact with experts from the Equinella team, a password-secured internal space within the online platform containing specific disease information sheets, reduced fees for laboratory diagnostic testing, and a mobile phone messaging service in case of an equine infectious disease outbreak in Switzerland (34).

Equinella data are stored in a database containing a total of 11 tables and can be exported in Microsoft Excel format. For the current analysis, data entered to Equinella since its initiation (November 23, 2013) until the April 26, 2019, were used.

### Data Validity

We assessed the data validity by using the handbook on data quality evaluation of monitoring and surveillance systems by the European Center for Disease Prevention and Control as a basis (36). Only variables from tables used for creating the output for this study were assessed for data validity. For the following parameters, this includes the tables “practice,” “reports,” and “laboratory.”

Technical data validity was assessed for the six parameters where comparable data within Equinella were available or where it was possible to set biological and logical ranges (Table 1): the number of equine patients of the practice, the percentage of equine workload, the number of veterinarians working in the practice, the date of practice registration to Equinella from the “practice” table, the date of finding from the “reports” table, and the laboratory diagnostic test used from the “laboratory” table. The approximate number of equine patients of the practice and entries for the percentage of equine workload was considered invalid if the values were zero. In addition, for practices of fewer than 10 animals or <5% equine workload, invalidity was assumed if the two parameters did not plausibly fit. The number of veterinarians within the practice was considered invalid if it was zero. The date when a practice registered to Equinella was considered invalid if the date entered was before November 2013. Similarly, the date of finding was considered invalid if it was before Equinella's online launch and if the date of report submission was before the date of findings. Because the date of report submission was automatically recorded by the system, it was taken as the correct date. The parameter of laboratory diagnostic test method was reviewed together with the variable on the reason for laboratory testing and suspected disease. If the suspected disease and the diagnostic test were biologically not compatible, the entry of the laboratory method was considered invalid. For example, if the diagnostic method was “bacteriology” and the suspected disease was a rotavirus infection, the entry was considered invalid.

**Table 1:** List of Equinella parameters assessed for data validity.

**Table name**	**Variable**	**Content**	**Data type**	**Validity % (valid/total entries)**
Reports				
	Date of findings	Date of the visit of the veterinarian and when the case was diagnosed by the veterinarian	Numerical	99.8 (629/630)
Practice				
	Veterinarians	Total number of veterinarians working in the practice	Numerical	95.7 (89/93)
	Equine workload in %	Percentage of equine workload of the practice	Numerical	90.3 (84/93)
	Number of animals	Approximate number of equine patients covered by the practice	Numerical	88.2 (82/93)
	Registration date	Date of the practice registering to Equinella	Numerical	100 (93/93)
Laboratory				
	Diagnostic method	Type of pathogen detection method	Categorical	99.8 (442/443)

### Descriptive Analysis of the Equinella Data

Descriptive analyses of the reports (clinical signs, diseases, age distribution, and laboratory diagnostics) were performed in either spatial or temporal context. Participating veterinarians and practices were described. Free text data entries such as for “other clinical signs” and “other diseases” were analyzed looking at the frequency of terms reported. Additionally, data on the age distribution of the Swiss equine population were drawn from a previous study (19) and were used to compare the age categories for strangles reports submitted to Equinella. All the analyses were conducted using the R statistical software version 3.5.1^3^.

### Timeliness of Reporting

The timeliness of a report was defined as the number of days between a veterinarian observing a diseased equine during a visit and reporting it on Equinella. A zero inflated negative binomial regression (ZINB) model was used to assess the change of the timeliness over time (37). The time in months from the initiation of Equinella (23rd of November 2013) until the end of the evaluation period (April 26, 2019) was used as a continuous independent variable in the model, and *p* < 0.05 for the model estimate was considered as significant.

### Activeness of Veterinarians and Confirmation of Equine Health

The activeness of veterinarians participating in Equinella was assessed by reviewing the number of reports per veterinarian for the entire evaluation period (between November 23, 2013, and April 26, 2019) and by providing an overview of active veterinarians of the most recent full calendar year, 2018. In addition, the frequency of monthly confirmed reminder emails (confirmation of no observed clinical case) was assessed for the veterinarians who never reported in 2018. Spearman correlation coefficient analysis was used to assess the association between the number of reports submitted per veterinarian and characteristics of the practice the veterinarian is working at (equine workload, number of equine patients, and number of overall and certified equine specialists at the respective practice). Results of this analysis are presented with the correlation coefficient (ρ) and considered as statistically significant when *p* < 0.05.

### Coverage of the Equine Population and Geographical Representativeness

During the registration process to Equinella, practices indicated how many equines are approximately counted within their patient registries. The equine population under surveillance by Equinella was defined as the sum of equines covered by all the practices registered to Equinella that have reported at least once during the evaluation period. The two university clinics Zurich and Bern mainly serve as referral clinics. Therefore, the equine patients of these two clinics were excluded from the calculations of the equine population under surveillance by Equinella. Coverage of the equine population was calculated by dividing the number of equines under surveillance by Equinella, by the total Swiss equine population. The latter derived from governmental census data obtained (April 2019) from the publicly accessible web portal. Registering equines to the governmental database AGATE is mandatory for every equine residing in Switzerland, and the database is updated monthly. Animal population demographical data entered to AGATE are made public through the government-hired company Identitas.

The geographical representativeness of the system was assessed by calculating the number of reports per 1,000 equines residing in each canton (member states of the Swiss confederation).

## Results

### Data Validity

Data validity in terms of data quality for all the six assessed parameters (“date of finding,” “veterinarians,” “equine workload in %,” “number of animals,” “registration date,” “diagnostic method”) ranged between 88.2 and 100% (Table 1). For variable “number of animals,” there was eight times an entry of zero. In two cases, it was reported that the number of animals was “1” combined with the entry of a workload of “70” and “100%,” and once, the number of animals was reported to be “2” with an equine workload of “50%.” These 11 entries were considered as invalid. In six cases, equine workload was reported to be “0%,” thus as well-considered as invalid.

### Descriptive Analysis of the Equinella Data

#### Equinella Reports

**Figure 1:**
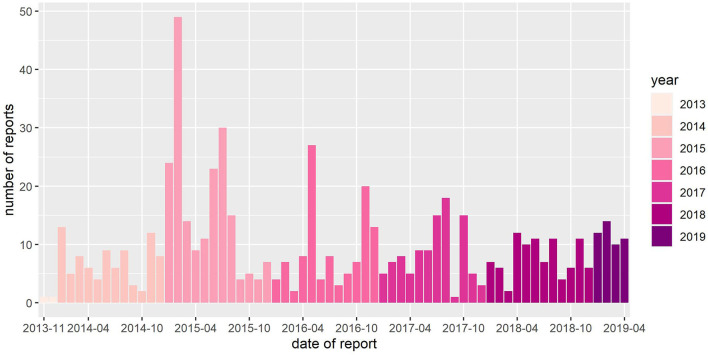
Number of reports submitted to Equinella between November 32, 2013, and April 26, 2019.

**Figure 2:**
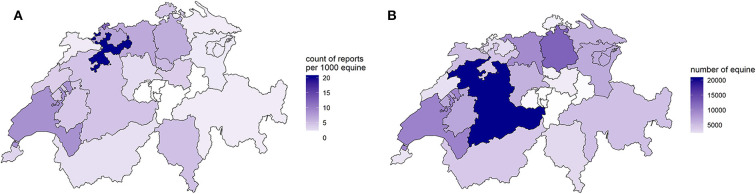
**(A)** Count of reports per 1,000 equine submitted to Equinella between November 23, 2013, and April 26, 2019, and **(B)** number of equine per canton from data originating from AGATE by April 2019.

Between November 23, 2013, and April 26, 2019, a total 630 reports were submitted to Equinella (Figure 1). Submitted reports originated from 21 of 26 Swiss cantons (Figure 2A). Considering the data reported from January 1, 2014, to December 31, 2018, the number of reports submitted to Equinella varied between 85 (2014) and 195 (2015) reports per year.

On the individual report basis, reports can include one or more clinical signs. Within the 630 reports, 721 clinical signs and 433 diseases were reported. Most reports recorded at least one clinical sign and one disease [*n* = 356 (56.5%)]. Other reports included a disease [*n* = 77 (12.2%)] or clinical signs [*n* = 197 (31.3%)] only.

**Figure 3:**
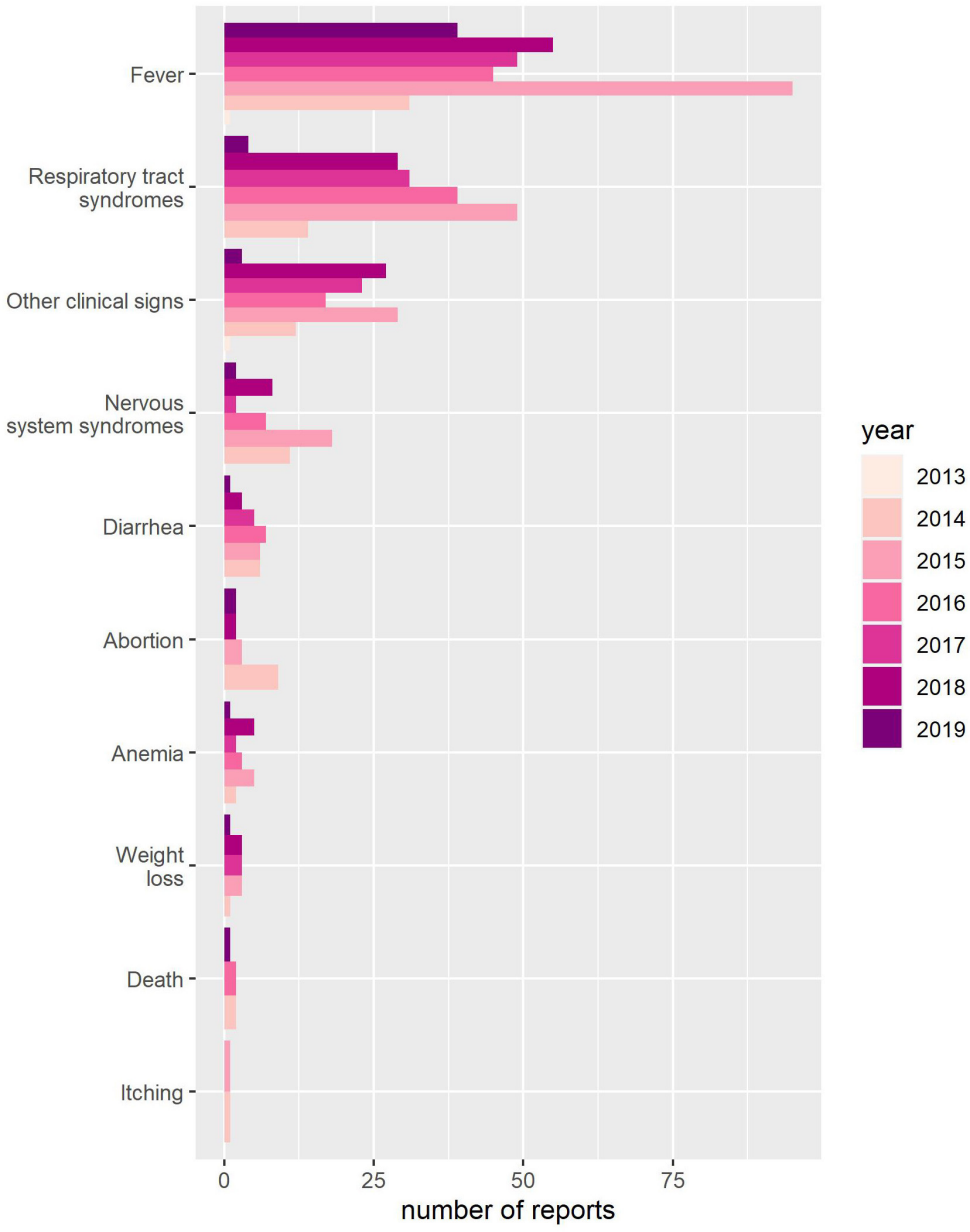
Yearly count of clinical signs reported to Equinella between November 23, 2013, and April 26, 2019.

Fever [*n* = 196 (31.1%)], respiratory tract syndromes [*n* = 94 (14.9%)], and other clinical signs [*n* = 56 (8.9%)] were the most frequently reported clinical signs among all reports (*n* = 630) (Figure 3). Of all reports with clinical signs (*n* = 553), the three most frequent combinations were fever and respiratory tract syndromes [*n* = 50 (9.0%)], fever and other clinical signs [*n* = 19 (3.4%)], and fever and nervous system syndromes [*n* = 16 (2.89%)]. Free text answers given to the option “other clinical signs” [*n* = 56) were most commonly colic [*n* = 12 (21.4%)], laryngitis [*n* = 8 (14.3%)], cough [*n* = 6 (10.7%)], and pharyngitis [*n* = 6 (10.7%)].

**Figure 4:**
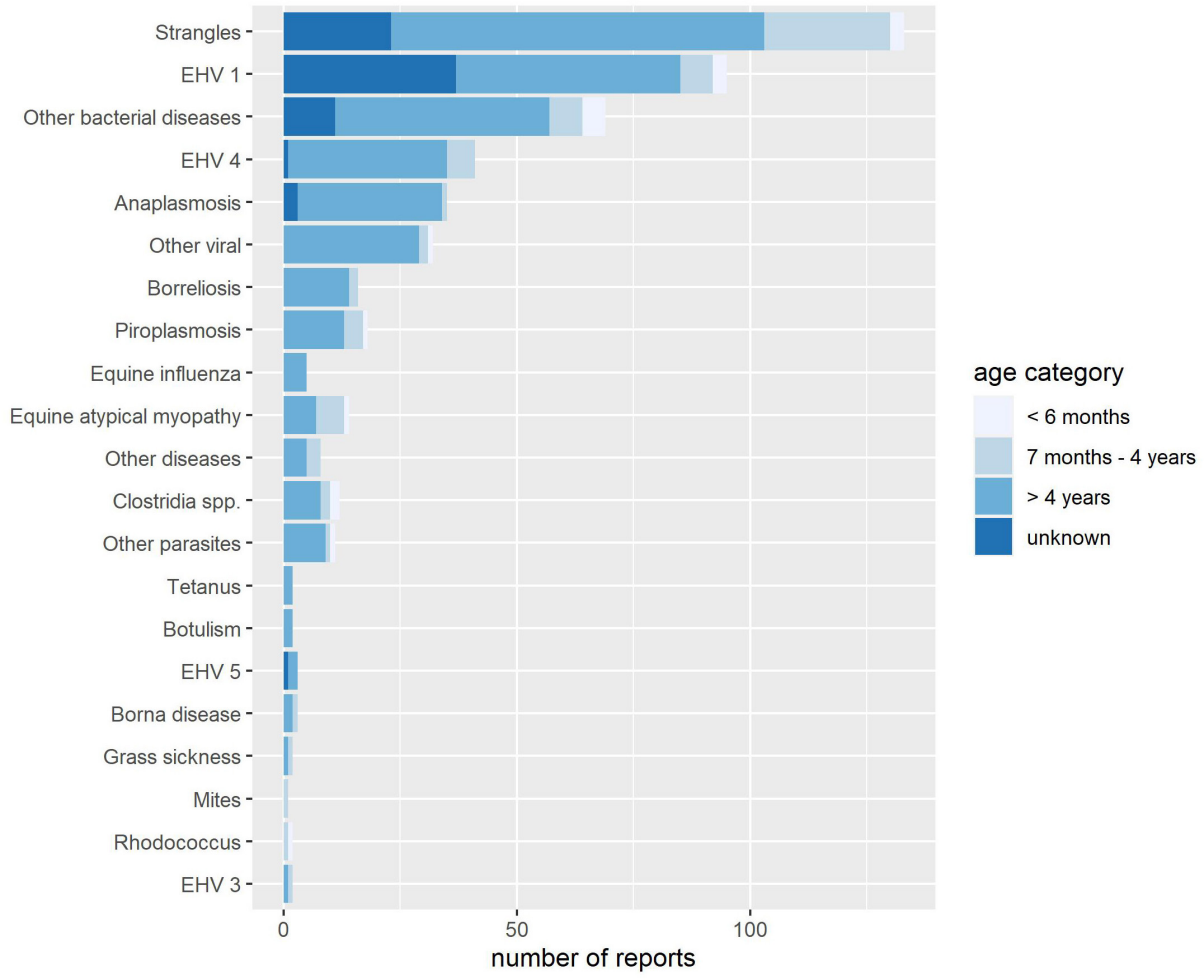
Count of diseases per age category reported to Equinella between November 23, 2013, and April 26, 2019.

Strangles [*n* = 127 (20.2%)], EHV-1 [*n* = 64 (10.2%)], and “other bacterial diseases” [*n* = 57 (9.1%)] were the most commonly reported diseases out of all reports (*n* = 630) (Figure 4). Reports from the “other” disease categories (*n* = 120) were most commonly other bacterial diseases [*n* = 69 (57.5%)]. The most frequent “other bacterial” disease reported was *Streptococcus equi* subspecies *equi* [*n* = 20 (29.0%)] followed by *S. equi* subspecies *zooepidemicus* [*n* = 12 (17.4%)]. Coronavirus [*n* = 7 (21.9%)] was the most frequently cited in free text among viral diseases (*n* = 32).

Of all reports (*n* = 630), the three most frequent combinations of disease and clinical sign were strangles and respiratory tract syndromes [*n* = 49 (7.8%)], EHV-1 and fever [*n* = 37 (5.9%)], and strangles together with respiratory tract syndromes and fever [*n* = 29 (4.6%)].

The majority of the 630 reports originate from equines categorized as more than 4 years [*n* = 451 (71.6%)], followed by equines of the age categories “unknown” [*n* = 82 (13.0%)], 7 months to 4 years [*n* = 79 (12.5%)], and 6 months or less [*n* = 18 (2.6%)] (Figure 4). Using the age distribution of the Swiss equine population as a denominator, the most common disease reported to Equinella, strangles had an incidence of 2.2 in 1,000 equines of 4 years or younger and 0.8 in 1,000 equines older than 4 years over the full evaluation period of 5.5 years. The incidence of strangles assessed by Equinella was thus found to be 2.75 times higher in young (≤ 4 years of age) equines compared to adult equines (>4 years of age).

#### Laboratory Diagnostics

In total, 443 laboratory tests were registered in Equinella and were associated with 346 reports (54.9% of all reports). The annual number of reports accompanied by a diagnostic test ranged from 34 (36.6% of all reports in 2014) to 61 (56.5% of all reports in 2017). Diagnostic tests were submitted most frequently for the following three diseases categories: “other diseases” [*n* = 111 (25.1%)], strangles [*n* = 102 (23.0%)] and EHV-1 [*n* = 70 (15.8%)] (Table 2). Of all submitted laboratory diagnostic results (*n* = 443), 293 (66.1%) stated to have confirmed the suspected disease.

**Table 2:** Results of the 10 most frequent diseases for which tests were submitted to Equinella between November 23, 2013, and April 26, 2019.

**Reported disease categories accompanied by a diagnostic testing**	**Total**	**Disease confirmed, n (%)**	**Disease not confirmed, n (%)**	**Test result pending, n (%)**
Other diseases	111	57 (51.4)	48 (43.2)	6 (5.4)
Strangles	102	80 (78.4)	21 (20.6)	1 (1.0)
EHV-1	70	44 (62.9)	24 (34.3)	2 (2.9)
EHV-4	42	28 (66.7)	14 (33.3)	—
Anaplasmosis	38	23 (60.5)	14 (36.8)	1 (2.6)
Piroplasmosis	19	13 (68.4)	5 (26.3)	1 (5.3)
Borreliosis	17	15 (88.2)	2 (11.8)	—
*Clostridia* spp.	14	12 (85.7)	2 (14.3)	—
Equine atypical myopathy	11	9 (81.2)	2 (9.1)	—
Borna disease	7	5 (71.4)	1 (14.3)	1 (14.3)

#### Equinella Veterinarians and Practices

In total, 102 veterinarians working in 93 different veterinary practices were registered within the Equinella database until April 2019 (Table 3). The median number of veterinarians working in a practice registered within Equinella was two (median), whereas this number ranged from 1 to 28. Overall, 24% of the veterinarians working in these practices were certified equine specialists; however, they are unequally distributed among the practices. The median number of self-reported equine patients per practice was 450 equines.

**Table 3:** Characteristics of the 93 practices and 102 veterinarians registered in Equinella system until April 2019.

**Practice data**	**Median**	**Range**
Number of veterinarians per practice	2	1–28
Number of certified equine specialist per practice	0	0–20
Number of equine patients per practice	450	10–10,000
**Veterinarian data**	**Count**	**Proportion %**
Veterinarians total	102	
Female	47	46.1
Male	55	53.9
German speaking	88	86.3
French speaking	14	13.7

### Timeliness of Reporting

Timeliness of reporting to Equinella ranged between 0 and 192 days (median, 7 days; interquartile range, 0–21 days). The ZINB model showed no association between the timeliness and the time since initiation of the online version of Equinella (*p* = 0.31322) (Supplementary Figure 1).

### Activeness of Veterinarians and Confirmation of Equine Health

During the entire evaluation period, 67 (65.7%) of the 102 veterinarians registered to Equinella have submitted a report at least once. From these 67 veterinarians, a median of 4 (range, 1–179) reports were received per veterinarian in 5.5 years. On average, 1.7 reports per veterinarian per year were submitted.

In 2018, 29 (28.4%) of 97 veterinarians who were registered until the end of the same year submitted at least one report. Of the 68 veterinarians who did not report in 2018, 59 (86.8%) have responded at least once to a monthly reminder email confirming absence of cases relevant for reporting to Equinella. The median number of monthly reminder responses for all non-reporting veterinarians in 2018 was nine (interquartile range, 4–11.5).

The number of reports submitted per veterinarian moderately correlated with the percentage of equine patient workload (ρ = 0.37, *p* = 0.004) and the total number of equine patients (ρ = 0.29, *p* = 0.027) at the practice of the respective reporting veterinarian. The number of reports submitted per veterinarian did not correlate with the overall number of veterinarians (ρ = 0.013, *p* = 0.92) working at the perspective practice. However, it moderately correlated with the number of certified equine specialist (ρ = 0.375, *p* = 0.003).

### Coverage of the Equine Population and Geographical Representativeness

Veterinary practices registered within Equinella that have reported at least once cover in total 59,835 equines, based on the practices' self-declaration on their number of equine patients. Taking the current size of the Swiss equine population as a denominator (112,466 equines, obtained from AGATE, April 30, 2019) (Figure 2B), Equinella covers 50.8% of the Swiss equine population. The incidences of submitted reports varied largely between cantons where equines are residing, with incidence between 0 and 20.3 reports per 1,000 equines and per canton within the past 5.5 years (Figure 2A).

## Discussion

In this article, we present data retrieved from over 5 years of equine surveillance by veterinarians on a voluntary basis in Switzerland. Collected data were reviewed in terms of data validity, timeliness, and representativeness, content of reports, and activeness of participating veterinarians.

The validity of data submitted to Equinella was regarded as high. The high data validity can be explained by a good collaboration between experts in information technology, veterinary public health, and equine health from government and universities during the creation and later maintenance of the surveillance tool. Such transdisciplinary collaborations allow a profound and well-thought-out data collection process. However, it was possible to validate only six parameters by using comparable internal data or setting biological or logical rules. Using external data (e.g., data driven from diagnostic laboratories receiving diagnostic samples from veterinarians registered to Equinella) would be beneficial to fully assess the validity of the Equinella data. In addition, some parameters such as the number of veterinarians working at practices registered to Equinella and the number of equine patients could be reassessed on a regular basis in order to have a more up-to-date information about participating practices and the coverage of the equine population. Unlike an usual assessment of data quality, at which data completeness would also be reviewed, this was not necessary for Equinella, because all of the data fields of value for the current analysis are either mandatory or have default settings.

Since November 2013, Equinella received on average 114 reports per year. In 2015, more reports were submitted compared to the other years. The peak in February 2015 was mainly caused by an increased number of reports of respiratory tract syndrome, fever, EHV-1, and strangles. This was due to an increased number of diseased equines from the same premises (confirmed by Equinella expert team) and also due to a one-time change in data entry management during February 2015. Occasionally, veterinarians contact the Equinella expert team to forward a certain report by phone call. Data entry is then conducted by the expert team in the name of the reporting veterinarian. Normally, the Equinella expert team enters such reports according to the definition of a report by the surveillance system; this means by reporting one diseased equine in a single report. However, in contrast to the report definition by the surveillance system and similar to the veterinarian's way of data entry in practice, in case that more than one equine is affected on the same premise during the same visit, the expert team enters the number of affected equines in the free text section. Because of a one-time employee change in February 2015, this way of data entry was handled differently. Multiple equines showing the same clinical signs, which were observed on the same premise and during one visit were entered individually in separate reports. This resulted in an increased number of reports, compared to the usual way of data entry management. Such non-standardized data entry procedures, in which the case definition of a report is not coherent with the reporting practice, do not only reduce the reliability of the surveillance system, but also they also impair the possibilities to use aberration detection algorithms for automatic early disease detection (38). Better harmonizing data entry procedures is critical to improve the assessment of the burden of non-notifiable equine infectious diseases in Switzerland and to offer the possibility moving toward the implementation of a syndromic surveillance system using automatic event detection (39). This can be achieved, first, by reassessing the objectives of the surveillance system and adjusting the case definition accordingly, and second, by offering participating veterinarians regular workshops and easy accessible standard operating procedure leaflets on correct data entry. Additionally, on the Equinella management level, submitted reports could be checked once a week for correct data entry and adjusted accordingly.

Strangles and EHV-1 were the diseases most frequently reported through Equinella. Strangles and EHV-1 are both diseases not notifiable by Swiss law, yet they are listed by the World Organization for Animal Health (OIE) as notifiable diseases (40). Capturing these two diseases through Equinella enables the veterinary officials in Switzerland to forward information to the OIE, leading to a contribution to the global surveillance of equine diseases.

Clinical signs, syndromes, and diseases reported under “other clinical signs” and “other diseases” are among the most frequently reported ones. Evaluating the content of free text field associated with these categories could help identifying clinical signs or diseases that have not been categorized yet. For example, *S. equi* subspecies *zooepidemicus*, an opportunistic commensal in equines and with zoonotic potential (41), is the second most frequently reported “other” disease. Adding it to the list of diseases in Equinella would help better monitoring this pathogen of public health interest. The analysis of the free text field also highlighted biases in the data reporting system. For example, the most commonly reported “other disease” was *S. equi* subspecies *equi* infection, the pathogen causing strangles (42). However, this should have been reported under the category “strangles” available in Equinella. This could lead to a potential underestimation of strangles cases due to false reporting. Regarding the “other clinical signs” most frequently reported, laryngitis and cough are commonly associated with respiratory tract infections, which should be reported under the already existing category of “respiratory tract syndromes.” Adding case definitions to clinical sign categories within the reporting tool and actively promoting them may therefore improve data reporting and therefore improve the estimation of correct reports.

Submitting a sample for diagnostic testing is not mandatory to submit a report to Equinella. Nevertheless, veterinarians have handed in samples for almost half of the reports submitted. This can be explained by certain diagnostic tests being subsidized by Equinella, the veterinarians' interest in following up their reports and the owner's willingness to pay for diagnostic testing. The majority (50.3%) of diagnostic testing was performed for “other diseases” as well as for strangles, highlighting the relevance of strangles in terms of giving precise recommendations to decrease disease transmission. Only a small proportion (2.9%) of laboratory diagnostic entries were neither ever confirmed nor declined (“test result pending”). All of these entries are dating back to diagnostics submitted between 2014 and 2016 and can therefore be expected to have obtained results but have not been updated within Equinella. To overcome this issue, laboratories could be enabled to enter laboratory test results for equine diseases directly into the Equinella database. Considering the limited time resources veterinarians have, this could also improve the timeliness of laboratory data submission. Nevertheless, this would demand resources for technical adaptation including a unique case identification. Also, the effort of including new stakeholders to a surveillance system should not be underestimated. On the Equinella management level, laboratory entries could be evaluated once a week allowing to identify and thereafter contact veterinarians where a laboratory test result is still pending. This could help maintaining continuously updated laboratory data.

Our results have shown that half (50.8%) of the equines in Switzerland are under observation by Equinella. However, this estimation of the coverage of the equine populations by Equinella comes along with some limitations. First, to calculate the number of equines under surveillance, we used the practices' statement at the time point of its registration to Equinella. This information was in most cases not updated thereafter, even if it might have changed over time. Second, to avoid an overestimation of population coverage, we excluded the university clinics for the calculation of equines under surveillance, because they are used as referral clinics, and patients might be counted twice if they are reported by both the practice and a referral clinic. However, there can be other referral clinics besides the university clinics that might contribute to an overestimation of the population coverage. For a more accurate assessment of the equine population coverage, the number of equine patients of all practices registered to Equinella could be reassessed on a regular basis.

Reports submitted to Equinella are mostly originating from cantons that have a higher density in equine population (Figure 2B). However, our study also highlighted differences in the representativeness of the Equinella surveillance system between cantons, assuming that equines from all cantons have the same risk of becoming diseased. As an example, cantons such as Vaud, Basel, and Solothurn are better represented, that is, have a higher incidence of reports, compared to central Switzerland (Figure 2A). To improve surveillance in the underreported regions, targeted awareness campaigns could be implemented to recruit more veterinarians working in such cantons. This might require an investigation of the system acceptability, such as assessing reasons why fewer reports are coming in from certain regions and whether incentives should be reevaluated. Transdisciplinary approaches aiming at structured effective collaborations across different disciplines and professions, such as stakeholder workshops including Equinella veterinarians, other equine veterinarians, equine veterinary associations, the Equinella team, and veterinary authorities may be a suitable format for such assessments.

The overall timeliness of the report submissions to Equinella was found to be 7 days in median. Studies evaluating public health surveillance system timeliness are in place yet rather not comparable (43). Data on timeliness from equine surveillance systems are scarce (44), making it rather difficult to compare the timeliness assed in Equinella. Timeliness of surveillance systems can depend on factors such as the format of data collection (paper vs. digital), type of incentives offered, and whether the reporting is mandatory or voluntary. An evaluation of a voluntary veterinary surveillance system for swine diseases in Canada has reported an average timeliness of 22.3 days, yet shorter timeliness (median of 1 day) and less variability with digital compared to paper-based data collection (45). Compared to the Canadian system, only digital data collection is provided within Equinella, yet the timeliness is still shorter.

Achieving high motivation for participation in voluntary surveillance systems is challenging, even though incentives are offered (46). Equinella offers incentives such as a monthly electronic newsletter on national and international equine health events, one free professional veterinary education course per year, and direct contact with experts from the Equinella team (34). Nevertheless, our analyses have revealed that approximately one-third of the veterinarians have registered to the system but never reported. In 2018, it was even fewer, with only one-third of all registered veterinarians submitting at least one report. The relatively low number of reports submitted by veterinarians can be explained by the fact that some veterinarians did not find the time to report; forgot to report, although reminded; or did not see an affected equine considered relevant enough to be reported. Despite the user-friendly web-based reporting tool in Equinella, reporting is an additional task during the typically busy schedule of a practicing veterinarian. Some of the registered veterinarians count only a few equines among their patients. Therefore, it is possible that not every veterinarian observes a relevant affected equine on a monthly basis, which is intended to be captured by the reminder emails. However, response to the reminder emails should also be improved to achieve a more constant confirmation of equine health and thus, to reliably evaluate absence of cases.

Although Equinella is designed to collect standardized data on defined clinical signs and diseases, the judgment of a veterinarian considering whether an affected equine is worth being reported or not depends on personal perception. This perception can also be driven by the perceived concern of equine owners calling veterinarians regarding certain diseases. The discrepancies between clinical signs and diseases as defined by a surveillance system and the actual reporting decision taken by the veterinarians can be improved by communicating the veterinarians' expectations toward and needs of the surveillance system more intensely. Keeping the participation high requires continuous feedback flow between voluntary participants and the surveillance system management team. This can be achieved by taking on a more transdisciplinary approach to surveillance system management, including conducting regular feedback dialogs accompanied by tailored incentives.

Because comparable data on the daily visit of veterinarians are currently not accessible, assessing the coverage of the reports submitted to Equinella compared to the number of real cases observed during the daily patient visits of a veterinarian is challenging Additionally, it can be expected that the number of patients a veterinarian encounters per day can greatly depend on the overall equine patient workload. Veterinarians who solely work in the equine field are more likely to see a higher number of equine patients compared to such that treat equines in addition to other animals. Our results suggest that the number of reports submitted per veterinarian positively correlates with the number of equine patients and equine workload of the practice the veterinarian is associated with. Furthermore, the same correlation was found for the number of certified equine specialists working at the respective practice, but not for the overall number of veterinarians. This suggests that indeed the more specialized a practice is, the more reports by their veterinarians are submitted. One possible way a more in-depth analysis could be is to request veterinarians to share their digital practice management tool entries in a confidential way. Reports of patients visits within such digital tools related to clinical signs and diseases that can also be reported on Equinella could potentially be used as a denominator for the cases reported to Equinella on veterinarian level.

A unique feature of the Equinella system is the reminder emails with the aim to collect information on the health of equines, by receiving the confirmation that no affected equine was observed during a full month. Responses to the Equinella reminder emails are also helpful to distinguish between the non-reporting but responsive veterinarians and the rest of the non-reporting veterinarians. When a veterinarian confirms that he/she did not observe any affected equine in the past month, the system tracks this information. This information can be used to assess the non-reporting but actively responding veterinarians. However, the system does not automatically trace if a veterinarian submits a report as a result of the reminder email. Such information would be valuable to get an estimation on the proportion of veterinarians that have forgotten to submit a report and would help to better assess the impact of reminders on data reporting.

As a veterinary-based surveillance system, Equinella has been used to monitor clinical signs and equine infectious diseases among the Swiss equine population since its online relaunch in November 2013. The surveillance system has been continuously receiving data since its initiation and has thus provided the national and international equine community with infectious disease insights from large parts of Switzerland. Reports submitted to Equinella have enabled obtaining an overview of the incidence of selected, non-notifiable diseases in the Swiss equine population. Additionally, practices registered to Equinella deliver valuable insights on what percentage of the equine population is under potential surveillance. The current Equinella data allow a comprehensive insight in health challenges and incidences of non-notifiable diseases of the Swiss equine population. Nevertheless, limitations of such voluntary surveillance systems should be regarded when interpreting surveillance data. The relatively low number of reports, variability in the veterinarian's participation, and their differing decision process whether to report a certain case make it challenging to determine the effective representativeness of the surveillance system. Improvement of such in regard to data consistency, harmonizing geographic coverage, and increasing the number of reports as well as the overall activeness of veterinarians must be pursued in collaboration with participating veterinarians by considering their capacities and needs. In this respect, fostering the veterinarians' contribution to the surveillance system through further targeted and regular collaborations, as well as using more transdisciplinary approaches, can help to improve toward a more representative, sustainable, and reliable veterinary-based voluntary surveillance system.

## Data Availability Statement

The datasets generated for this study will not be made publicly available because they contain confidential surveillance data such as addresses, names and e-mail addresses. Requests to access the datasets should be directed to Equinella, info@equinella.ch.

## Author Contributions

RÖ, FR-W, CF, and SD contributed to the conception and design of the study. RÖ and FR-W provided access to data. RÖ wrote the first draft of the manuscript and organized the data. RÖ, CF, and SD performed the statistical analysis. All authors contributed to the data interpretation, manuscript revision, read, and approved the submitted version.

## Conflict of Interest

The authors declare that the research was conducted in the absence of any commercial or financial relationships that could be construed as a potential conflict of interest.

## Footnotes

^1^National Equine Health Survey, UK, https://www.bluecross.org.uk/national-equine-health-survey-0 (accessed January 10, 2020).

^2^Equinella, www.equinella.ch (accessed January 10, 2020).

^3^R statistical software version 3.5.1, https://www.r-project.org/ (accessed January 10, 2020).

## References

[1] Balasuriya UBR Carossino M Timoney PJ. Equine viral arteritis: a respiratory and reproductive disease of significant economic importance to the equine industry. Equine Vet Educ. (2018) 30:497–512. 10.1111/eve.1267225441113

[2] Broaddus CC Balasuriya UBR Timoney PJ White JLR Makloski C Torrisi K. Infection of embryos following insemination of donor mares with equine arteritis virus infective semen. Theriogenology. (2011) 76:47–60. 10.1016/j.theriogenology.2011.01.017 21345485

[3] Roberts H. Equine infectious anaemia in Europe: an ongoing threat to the UK. Vet Rec. (2017) 181:442–6. 10.1136/vr.j4721 29074793

[4] Waller AS. Strangles: taking steps towards eradication. Vet Microbiol. (2013) 167:50–60. 10.1016/j.vetmic.2013.03.033 23642414

[5] Sack A Cullinane A Daramragchaa U Chuluunbaatar M Gonchigoo B Gray GC Equine influenza virus- a neglected, reemergent disease threat. Emerg Infect Dis. (2019) 25:1185–91. 10.3201/eid2506.161846

[6] Smyth G Dagley K. Internet-based survey of horse owners for mortality and morbidity related to equine influenza in the 2007 Australian epidemic. Aust Vet J. (2011) 89:23–5. 10.1111/j.1751-0813.2011.00776.x 21711277

[7] Smyth G Dagley K Tainsh J. Insights into the economic consequences of the 2007 equine influenza outbreak in Australia. Aust Vet J. (2011) 89:151–8. 10.1111/j.1751-0813.2011.00777.x 21711317

[8] Weese JS Infection control and biosecurity in equine disease control. Equine Vet J. (2014) 46:654–60. 10.1111/evj.12295 24802183 PMC7163522

[9] Taylor M Agho K Stevens G Raphael B. Factors associated with high psychological distress in horse industry participants during the 2007 Australian equine influenza outbreak and evidence of recovery after 1 year. Aust Vet J. (2011) 89:158–9. 10.1111/j.1751-0813.2011.00772.x 21711318

[10] Morbidity and Mortality Weekly Report (2002). Available online at: https://www.ncbi.nlm.nih.gov/pmc/articles/PMC5657660/pdf/mm6602a3.pdf (accessed July 24, 2019).

[11] Leon CA Jaramillo R Martinez S Fernandez F Téllez H Lasso B. Sequelae of venezuelan Equine Ecephalitis in humans: a four year follow-up. Int J Epidemiol Oxford Univ Press. (1975) 4:131–41. 10.1093/ije/4.2.131 1165151

[12] Playford EG McCall B Smith G Slinko V Allen G Smith I. Human hendra virus encephalitis associated with Equine outbreak, Australia, 2008. Emerg Infect Dis. (2010) 16:219–23. 10.3201/eid1602.090552 20113550 PMC2957996

[13] Weese JS A review of Equine zoonotic diseases: risks in veterinary medicine. In: AAEP Procedings. Orlando (2002). p. 362–70.

[14] Young JJ Coulombier D Domanović D Zeller H Gossner CM. One health approach for West Nile virus surveillance in the European Union: relevance of equine data for blood safety. Eurosurveillance. (2019) 24: 1800349. 10.2807/1560-7917.ES.2019.24.16.1800349 31014416 PMC6826348

[15] European Center for Disease Prevention and Control Communicable Disease Threats Reports. (2019). Available online at: https://www.ecdc.europa.eu/en/threats-and-outbreaks/reports-and-data/weekly-threats (accessed June 17, 2020).

[16] Campbell GL Marfin AA Lanciotti RS Gubler DJ West Nile virus. Lancet Infect Dis. (2002) 2:519–29. 10.1016/S1473-3099(02)00368-7 12206968

[17] Porter RS Leblond A Lecollinet S Tritz P Cantile C Kutasi O. Clinical diagnosis of west nile fever in equids by classification and regression tree (CART) analysis and comparative study of clinical appearance in three European countries. Transbound Emerg Dis. (2011) 58:197–205. 10.1111/j.1865-1682.2010.01196.x 21208395

[18] Andersson K Lehtola M Regulating the new equine industry in finland. Wicked problems, governance models and gendered power structures. Sociol Ruralis. (2011) 51:387–403. 10.1111/j.1467-9523.2011.00545.x

[19] Schmidlin L Bachmann I Flierl S Schwarz A Roesch A Rieder S Die Schweizer Pferdebranche (2014).

[20] Reynaud E Niederhäusern R von Ackermann C Das Arbeitspferd in der Schweiz Erhebung 2017 (2018). Available online at: https://www.agroscope.admin.ch/agroscope/de/home/publikationen/suchen/agroscope-transfer.html (accessed June 17, 2020).

[21] Herholz C Füssel AE Timoney P Schwermer H Bruckner L Leadon D. Equine travellers to the olympic games in Hong Kong 2008: a review of worldwide challenges to equine health, with particular reference to vector-borne diseases. Equine Vet J. (2008) 40:87–95. 10.2746/042516408X253136 18083666

[22] Dominguez M Münstermann S de Guindos I Timoney P. Equine disease events resulting from international horse movements: systematic review and lessons learned. Equine Vet J. (2016) 48:641–53. 10.1111/evj.12523 26509734

[23] Metcalf ES. The role of international transport of equine semen on disease transmission. Anim Reprod Sci. (2001) 68:229–37. 10.1016/S0378-4320(01)00159-2 11744267

[24] Timoney PJ Infectious diseases and international movement of horses. In: Equine Infectious Diseases. Elsevier (2014). p. 544–51.e1. 10.1016/B978-1-4557-0891-8.00063-4

[25] Vanderman KS Swinker AM Gill BE Radhakrishna RB Kniffen DM Staniar WB Survey on the implementation of National equine identification in the United States. J Equine Vet Sci. (2009) 29:819–22. 10.1016/j.jevs.2009.10.014

[26] Murray G Munstermann S Lam K Benefits and Challenges Posed by the Worldwide Expansion of Equestrian Events – New Standards for the Population of Competition Horses and Equine Diseases Free Zones (EDFZ) in Countries. (2013). Available online at: http://citeseerx.ist.psu.edu/viewdoc/download?doi=10.1.1.444.2561&rep=rep1&type=pdf (accessed June 17, 2020).

[27] Slater J. Equine disease surveillance: findings from the national equine health survey, 2013. Vet Rec. (2014) 175:271–2. 10.1136/vr.g4982 25234456

[28] Slater J Taylor G National Equine Health Surveillance. Blue Cross for Pets. United Kingdom (2018). Available online at: https://www.bluecross.org.uk/sites/default/files/downloads/NEHS-results-2018.pdf

[29] Faverjon C Vial F Andersson MG Lecollinet S Leblond A. Early detection of West Nile virus in France: quantitative assessment of syndromic surveillance system using nervous signs in horses. Epidemiol Infect. (2017) 145:1044–57. 10.1017/S0950268816002946 27938434 PMC9507807

[30] Ackermann C Rieder S von Niederhäusern R Kennzahlen der Schweizer Pferdebranche Teil I : Equidenbestand und Seine Zusammensetzung. (2018). Available online at: https://www.agroscope.admin.ch/agroscope/de/home/publikationen/suchen/_jcr_content/par/externalcontent.external.exturl.pdf/aHR0cHM6Ly9pcmEuYWdyb3Njb3BlLmNoL2ZyLUNIL0FqYXgvRW/luemVscHVibGlrYXRpb24vRG93bmxvYWQ_ZWluemVscHVibGlr/YXRpb25JZD0zODU3MQ==.pdf (accessed June 17, 2020).

[31] Federal Council of Switzerland Ordinance on Epizootic Diseases. (1995). Available online at: https://www.admin.ch/opc/de/classified-compilation/19950206/index.html (accessed June 17, 2020).

[32] Meier HP Hauser R. The monitoring of infectiuous diseases in Switzerland. Pferdeheilkunde. (1996) 12:569–70. 10.21836/PEM1996044626799652

[33] Wohlfender FD Schüpbach G Gerber V Wehrli Eser M Hauser R Meier HP A review of twenty years of equine infectious disease monitoring in Switzerland: past, present and future. J Equine Vet Sci. (2012) 32:S92 10.1016/j.jevs.2012.08.196

[34] Struchen R Hadorn D Wohlfender F Balmer S Süptitz S Zinsstag J. Experiences with a voluntary surveillance system for early detection of equine diseases in Switzerland. Epidemiol Infect. (2016) 144:1830–6. 10.1017/S0950268816000091 26846449 PMC9150615

[35] Wohlfender-Remy F Struchen R Graubner C Balmer S Hadorn D Re-launch of Equinella: a web-based equine disease reporting and information platform. J Equine Vet Sci. (2016) 39:S17 10.1016/j.jevs.2016.02.035

[36] European Centre for Disease Prevention and Control Data Quality Monitoring and Surveillance System Evaluation: A Handbook of Methods and Applications. ECDC, 2014. (2014). Available online at: https://www.ecdc.europa.eu/en/publications-data/data-quality-monitoring-and-surveillance-system-evaluation-handbook-methods-and (accessed June 17, 2020).

[37] Jackman S Tahk A Zeileis A Maimone C Fearon J Maintainer ZM Political Science Computational Laboratory Version 1.5.2, R Package “pscl”. (2017).

[38] Vial F Berezowski J. A practical approach to designing syndromic surveillance systems for livestock and poultry. Prev Vet Med. (2015) 120:27–38. 10.1016/j.prevetmed.2014.11.015 25475688

[39] Faverjon C Berezowski J. Choosing the best algorithm for event detection based on the intend application: a conceptual framework for syndromic surveillance. J Biomed Inform. (2018) 85:126–35. 10.1016/j.jbi.2018.08.001 30092359

[40] OIE International Animal Health Code. (1968). Available online at: http://www.oie.int (accessed January 24, 2020).

[41] Pelkonen S Lindahl SB Suomala P Karhukorpi J Vuorinen S Koivula I. Transmission of *streptococcus equi* subspecies zooepidemicus infection from horses to humans. Emerg Infect Dis. (2013) 19:1041–8. 10.3201/eid1907.121365 23777752 PMC3713971

[42] Boyle AG Strangles and its complications. Equine Vet Educ. (2017) 29:149–57. 10.1111/eve.12568

[43] Jajosky RA Groseclose SL. Evaluation of reporting timeliness of public health surveillance systems for infectious diseases. BMC Public Health. (2004) 4:29. 10.1186/1471-2458-4-29 15274746 PMC509250

[44] Faverjon C Andersson MG Decors A Tapprest J Tritz P Sandoz A. Evaluation of a multivariate syndromic surveillance system for West nile virus. Vector Borne Zoonotic Dis. (2016) 16:382–90. 10.1089/vbz.2015.1883 27159212 PMC4884334

[45] del Rocio Amezcua M Pearl DL Friendship RM McNab WB. Evaluation of a veterinary-based syndromic surveillance system implemented for swine. Can J Vet Res. (2010) 74:241–51. 21197223 PMC2949336

[46] Cowled B Ward MP Hamilton S Garner G. The equine influenza epidemic in Australia: spatial and temporal descriptive analyses of a large propagating epidemic. Prev Vet Med. (2009) 92:60–70. 10.1016/j.prevetmed.2009.08.006 19748691

