# Supplementary material for: Evaluating 5.5 Years of Equinella: A Veterinary-Based Voluntary Infectious Disease Surveillance System of Equines in Switzerland
Source: Front Vet Sci. 2020 Jun 30;7:327. doi: 10.3389/fvets.2020.00327 (PMC7339941; doi:10.3389/fvets.2020.00327)
Supplement: Supplementary file 1 [file Data_Sheet_1.docx]

Supplementary material with
"Evaluating 5.5 years of Equinella: a veterinary-based voluntary infectious disease surveillance system of equines in Switzerland"

| African horse sickness |
| --- |
| Vesicular stomatitis |
| Dourine |
| Equine infectious anemia |
| Anthrax |
| Glanders |
| Rabies |
| Tuberculosis |
| Contagious equine metritis |
| Leptospirosis |
| Equine encephalomyelitis (Wester, Eastern, Venezuelan) |
| Salmonellosis |
| West-Nile Fever |
| Cryptosporidiosis |
| Equine viral arteritis |
| Toxoplasmosis |
| Trichinellosis |
|  |

**Supplementary Table 1.** List of equine diseases notifiable according to the Swiss animal health law (Tierseuchenverordnung 916.401) from 1966 (<https://www.admin.ch/opc/de/classified-compilation/19950206/index.html>)

**
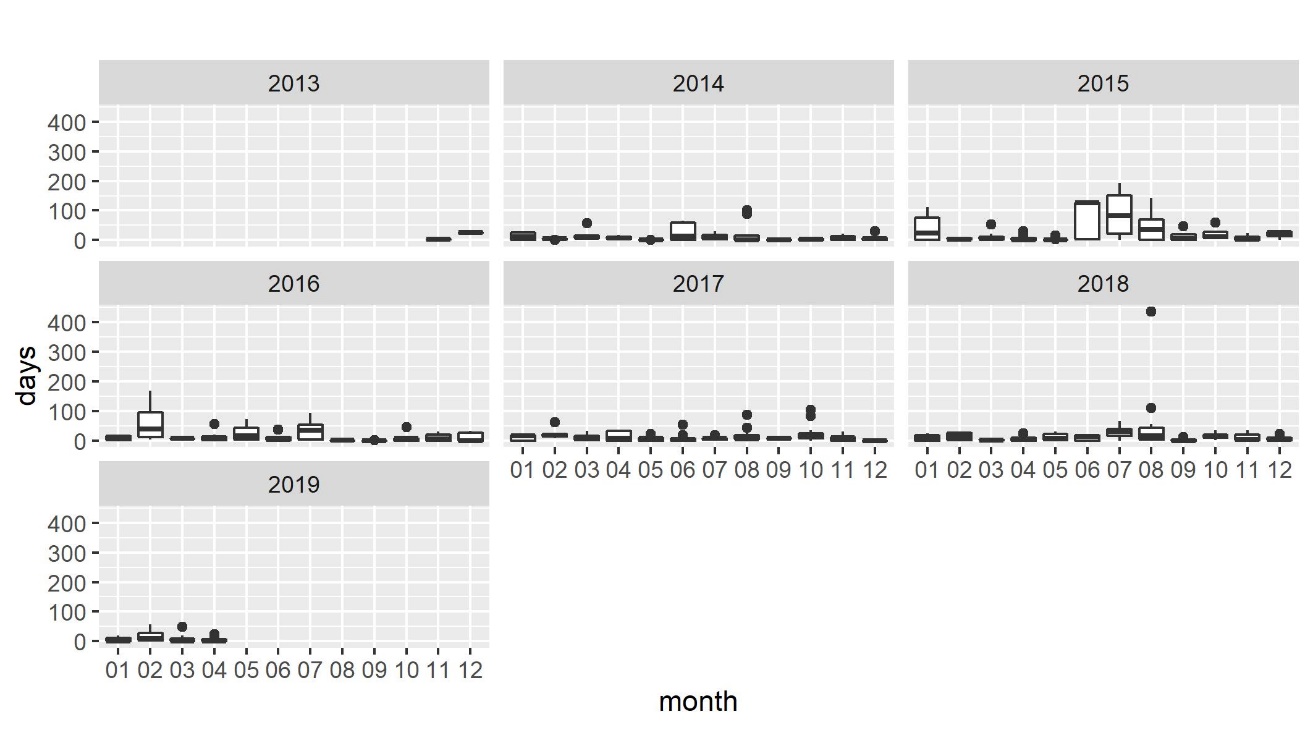
**

**Supplementary Figure 1.** Median timeliness (days between the date of findings and date of report submission) of reports submitted to Equinella between November 23, 2013, and April 26, 2019, per month.
